# Eco-Contribution for the Production of *N*-Arylnitrones: Solvent-Free and Assisted by Microwaves

**DOI:** 10.3390/ijms11062576

**Published:** 2010-06-22

**Authors:** Leonor Reyes, Sandra Corona, Gabriel Arroyo, Francisco Delgado, René Miranda

**Affiliations:** 1 Departamento de Ciencias Químicas, Facultad de Estudios Superiores Cuautitlán-UNAM, Estado de México, 54754, Mexico; E-Mails: lrdcistrans@yahoo.com.mx (L.R.); sandy_corjua@yahoo.com.mx (S.C.); garroyo@unam.mx (G.A.); 2 Departamento de Química Orgánica, Escuela Nacional de Ciencia Biológicas-IPN, México D.F., 11340, Mexico; E-Mail: fdelgado@woodward.encb.ipn.mx (F.D.)

**Keywords:** green chemistry, arylnitrones, microwave, solvent-less, catalyst-less

## Abstract

A simple green approach for the production of benzylideneaniline oxides is offered. This contribution was performed via the condensation of phenylhydroxylamine with several aryl aldehydes, in the absence of both catalyst and solvent, and using microwave irradiation as the activating reaction mode. In addition, good yields of the products were achieved in a short time. It is also worth noting that the work-up procedure is simple and the products do not require further purification. Finally, an interesting comparison without the use of microwave irradiation is also discussed.

## 1. Introduction

Microwave provides a powerful way in synthesis in light of the green chemistry protocol; in other words, it furnishes many chemical reaction improvements, such as enhanced reaction rates, higher yields of pure products as well as eco-friendly advantages [1]. Thus, procedures employing microwave methodology involving an appropriate green-approach must be welcome. In a different way, benzylideneaniline oxides commonly known as arylnitrones are highly valuable synthetic intermediates [2] and excellent spin trapping reagents [3,4]. These compounds are of high interest due to their wide spectra as synthons in the construction of natural products of the class of alkaloids [5]. This, in addition to the 1,3-dipolar cycloaddition of nitrones with acetylenic or olefinic dipolarophiles yielding highly substituted isoxazolidines and isoxazole derivatives with generation of, as many as, three new contiguous stereogenic centers in a single step [6,7]. Nitrones, the target molecules of this work, have been prepared using many methods, for example: by the condensation of carbonyl compounds with the corresponding hydroxylamines in refluxing CH_2_Cl_2_ in a suspension of anhydrous MgS0_4_ [ 8]; via the oxidation of secondary amines or *N*,*N*′-disubstituted hydroxylamines [9,10]; also by the oxidation of secondary amines with hydrogen peroxide in the presence of catalytic amounts of sodium tungsten [11–13]; by direct condensation of β-hydroxylamine alcohols with *ortho*-esters or amide acetals [14]; the catalytic oxidation of imines to nitrones [15] the condensation of equimolar amounts of aldehydes and *N*-substituted-hydroxylamines under solvent-free conditions in a ball-mill [16]. It is important to note that several methyl and *ter*-butyl nitrones have also been obtained employing microwaves, but using the substrates as their corresponding hydrochloride, requiring in addition the presence of sodium acetate [17]. Even throughout the good to excellent results that could be achieved in all these cases, all of them show limitations: the use of organic solvents, relatively long reaction times, the use of an excess of the aldehyde to achieve high conversion, tedious chromatographic purification in addition to environmentally non-benign oxidants with metal catalysts. In this sense, and taking into account the importance of the alkylnitrones, new methods to produce the title product are always welcome.

As part of our research program, we are interested in the generation of novel methods, with an appropriate green approach, for the production of organic molecules of particular interest [18–22]. Thereby, the goal of this work is to offer of a novel method with a green approach in order to produce various nitrones (benzylideneaniline oxides), via condensation of phenylhydroxylamine with several aryl aldehydes, in the absence of catalyst and solvent, and moreover using microwave irradiation, for the target purpose, as the activating reaction mode.

## 2. Results and Discussion

**Scheme 1.**
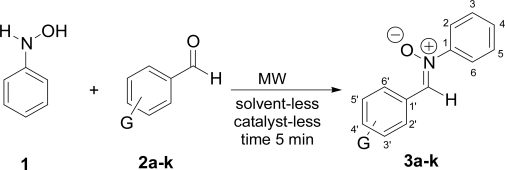
Synthesis of benzylideneaniline oxides (**3a–k**).

In view of the potential importance of arylnitrones, and considering the limitations of existing methods, a green contribution is presented in this work. The method involves the microwave irradiation of a mixture of phenylhydroxylamine (1) and an aromatic aldehyde (**2a–k**) under solvent-free conditions and, moreover, without an environmentally non-benign catalyst (Scheme 1). The target is to produce in a stereoselectively form a series of eleven benzylideneaniline oxides (**3a–k**). The corresponding condensation results are summarized in Table 1. As can be seen, the target molecules were produced in a very short time, in comparison to those methods previously reported, with good yields. The products were achieved with good purity after ethanol washing of the reaction mixture. Consequently, this procedure offers a simple work up.

It is important to note that in order to have an appropriated comparison, the corresponding thermal reactions were also performed using a heating mantle (neat, without catalyst, same time and temperature). However, according to the respective proton NMR data (aldehydic *vs.* vinilic proton), no more than 30–50% of substrate transformation was achieved.

For the structural attribution of the obtained compounds, common spectroscopic methods were employed. In this sense, after purification, the analysis of ^1^H-NMR spectra of compounds **3a**–**k** allowed us to conclude that they really have a similar structure. The resonance of proton at the nitrone group carbon N=CH appears as a singlet between δ 7.86–8.77 ppm. H-2 is also more deshielded because there is one group nitro in the aromatic ring **3c**, **3d**, **3g** or when the group is in the *ortho* position **3b**, **3j**. In the ^13^C-NMR spectra of the nitrones **3a**–**k,** the signal of the methine carbon of the imine function appeared at δ 132–134 ppm.

In regard to the EIMS, in general the products exhibit a set of three common peaks, corresponding to the expected molecular ions M^+^, in addition to [M-1]^+^ and [M-17]^+^ due to loss of hydrogen and a hydroxyl group, respectively, between others peaks of particular interest.

**Figure 1:**
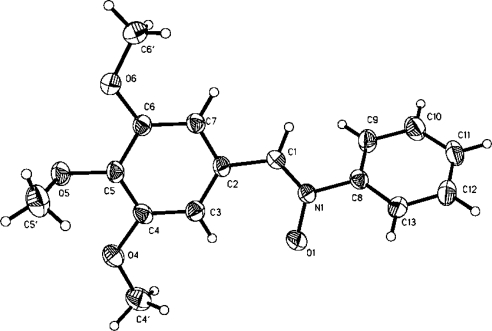
ORTEP of nitrone **3i**.

Finally, it is important to note that the *Z*-configuration of the obtained products was inferred by the X-ray, single crystal, diffraction of compound **3i**, according to the corresponding Oak Ridge Thermal Ellipsoid Plot (ORTEP), Figure 1.

## 3. Experimental Section

Melting points (uncorrected) were determined with a Fisher-Johns apparatus. ^1^H and ^13^C spectra were recorded on Varian (200 MHz), Varian Mercury (300 MHz) and Varian VNMR System (500 MHz) NMR instruments, with DMSO-d_6_ o CDCl_3_ as the solvent and TMS as internal standard. Mass spectra (MS) were recorded, in electron impact mode, with Hewlett–Packard 5971A and Thermo-Finnigan Polaris Q spectrometers. X-ray crystallographic structure was obtained on Siemens P4 diffractometer. Analytical TLC was carried out using E. Merck silica gel 60 F254 coated 0.25 plates, visualized by a long- and short-wavelength UV lamp. Flash column chromatography was performed over silica gel (230–400 mesh). All reagents, as well as solvents, were of reagent grade from Aldrich and were used without further treatment. Phenylhydroxylamine **1** was prepared as previously reported [23,24].

**General Method:** A mixture of phenylhydroxylamine **1** (0.5 mmol) and the corresponding aromatic aldehyde **2a**–**k** (0.5 mmol) were irradiated with microwave at 80 °C and 60 W during 5 min. Finally, the crude product was filtered and the solid obtained was recrystallized from ethanol to give the pure nitrones **3a–k**.

**(*Z*)-*N*-(3-hydroxybenzylidene) aniline oxide (3a).** 76% yield; yellow solid, Mp 95–96 °C; ^1^H NMR (200 MHz, DMSO*_d-6_*): δ 9.67 (s, 1H, OH), 8.41 (s, 1H, N=CH), 8.17 (s, 1H, H-2’), 7.92–7.88 (m, 2H, H-2 and H-6), 7.72 (d, *J* = 7.4 Hz, 1H, H-6’), 7.58-7.52 (m, 3H, H-3, H-4 and H-5), 7.29 (t, *J* = 7.4 Hz, 1H, H-5’), 6.91 (d, *J* = 8.2 Hz, 1H, H-4’); ^13^C*-*NMR (50 MHz, DMSO*_d-6_*): δ 157.1 (C-3’), 148.5 (C-1), 133.7 (C-2’), 132.0 (N=CH), 129.8 (C-4), 129.1 (C-3 and C-5), 121.5 (C-2 and C-6), 120.6 (C-6’), 117.9 (C-4’), 114.7 (C-1’); EIMS (70 eV) m/z (% relative abundance): 198 (28) [M-OH]^+^, 169 (100) [M-CO_2_H]^+·^.

**(*Z*)-*N*-(2-hydroxybenzylidene) aniline oxide (3b).** 85% yield; yellow solid Mp 147–148 °C; ^1^H-NMR (500 MHz, DMSO*_d-6_*): δ 12.51 (s, 1H, OH), 8.72 (s, 1H, N=CH), 7.94-7.92 (m, 2H, H-2 and H-6), 7.76 (d, 8 Hz, 1H, H-6’), 7.58-7.54 (m, 3H, H-3, H-4 and H-5), 7.43 (t, J = 8 Hz, 1H, H-4’), 6.93-6.88 (m, 2H, H-3’ and H-5’); ^13^C-NMR (125 MHz, DMSO*_d-6_*): δ 158.7 (C-2’), 145.8 (C-1), 139.9 (C-4’), 134.1 (N=CH), 133.0 (C-6’), 130.1 (C-4), 129.1 (C-3 and C-5), 121.6 (C-2 and C-6), 118.7 (C-3’), 118.6 (C-5’), 117.3 (C-1’); EIMS (70 eV) m/z (% relative abundance): 198 (28) [M-OH]^+^, 169 (100) [M-CO_2_H]^+·^.

**(*Z*)-*N*-(4-nitrobenzylidene) aniline oxide (3c).** 80% yield; yellow solid, Mp 120 °C; ^1^H-NMR (500 MHz, CDCl_3_): δ 8.36 (d, 2H, *J* = 8.5 Hz, H-2’ and H-6’), 7.90 (s, 1H, N=CH), 7.77-7.75 (m, 2H, H-2 and H-6), 7.49-7.47 (m, 3H, H-3, H-4 and H-5), 7.44 (d, *J* = 8.5 Hz, 2H, H-3’ and H-5’); ^13^C-NMR (125 MHz, CDCl_3_): δ 148.9 (C-1), 136.3 (C-4’), 133.3 (N=CH), 130.1 (C-2’, C-6’), 130.0 (C-4), 129.2 (C-3, C-5), 129.1 (C-1’), 128.9 (C-3’ and C-5’), 121.6 (C-2 and C-6); EIMS (70 eV) m/z (% relative abundance): 226 (100) [M-O]^+^, 225 (50) [M-OH]^+·^.

**(*Z*)-*N*-(2-nitrobenzylidene) aniline oxide (3d).** 77% yield; yellow solid, Mp 85 °C; ^1^H-NMR (300 MHz, DMSO*_d-6_*): δ 8.77 (s, 1H, N=CH), 8.44 (d, *J* = 12.5 Hz, 1H, H-6’), 8.10 (d, *J* = 12.5 Hz, 1H, H-3’), 7.93-7.90 (m, 1H, H-5’), 7.90-7.83 (m, 2H, H-2 and H-6), 7.71 (t, *J* = 12.5 Hz, 1H, H-4’), 7.60-7.56 (m, 3H, H-3, H-4 and H-5); ^13^C-NMR (75 MHz, DMSO*_d-6_*): δ 147.9 (C-2’), 147.7 (C-1), 133.2 (N=CH), 130.7 (C-6’), 130.5 (C-5’), 130.3 (C-4), 129.4 (C-4’), 129.2 (C-3 and C-5), 124.4 (C-3’), 124.1 (C-1’), 121.4 (C-2 and C-6); EIMS (70 eV) m/z (% relative abundance): 242 (5) [M]^+·^, 141 (4) [M-1]+·, 226 (100) [M-O]^+^, 225 (50) [M-OH]^+·^.

**(*Z*)-*N*-(4-methoxybenzylidene) aniline oxide (3e).** 86% yield; yellow solid, Mp 108 °C; ^1^H-NMR (500 MHz, CDCl_3_): δ 8.41 (d, *J =* 8.5 Hz, 2H, H-2’ and H-6’), 7.86 (s, 1H, N=CH), 7.77 (d, *J =* 8.8 Hz, 2H, H-2 and H-6), 7.47-7.43 (m, 3H, H-3, H-4 and H-5), 6.98 (d, *J* = 8.5 Hz, 2H, H-3’ and H-5’); ^13^C-NMR (125 MHz, CDCl_3_): δ 161.1 (C-4’), 148.4 (C-1), 133.7 (N=CH), 130.7 (C-6’ and C-2’), 129.2 (C-4), 128.7 (C-3 and C-5), 123.3 (C-1’), 121.2 (C-2 and C-6), 113.6 (C-3’ and C-5’), 55.0 (OCH_3_); EIMS (70 eV) m/z (% relative abundance): 227(50) [M]^+·^, 226 (80) [M-1]^+^, 210 (100) [M-OH]^+^.

**(*Z*)-*N*-(benzylidene) aniline oxide (3f).** 81% yield; yellow solid, Mp 130 °C; ^1^H-NMR (300 MHz, CDCl_3_): δ 8.42-8.38 (m, 2H, H-2’ and H-6’), 7.92 (s, 1H, N=CH), 7.79-7.76 (m, 2H, H-2, H-6), 7.50-7.46 (m, 6H, H-3, H-4, H-5, H-3’, H-4’ and H-5’); ^13^C-NMR (125 MHz, CDCl_3_): δ 149.0 (C-1), 134.6 (N=CH), 130.9 (C-4), 130.5 (C-1’), 129.9 (C-4’), 129.1 (C-3’ and C-5’) 129.0 (C-3 and C-5), 128.6 (C-2’ and C-6’), 121.7 (C-2 and C-6); EIMS (70 eV) m/z (% relative abundance): 197 (35) [M]^+·^, 196 (60) [M-1]^+^, 180 (175) [M-OH]^+^, 181 (50) [M-O]^+·^.

**(*Z*)-*N*-(3-nitrobenzylidene) aniline oxide (3g).** 68% yield; yellow solid, Mp 110 °C; ^1^H-NMR (300 MHz, CDCl_3_): δ 9.19 (s, 1H, H-2’), 8.83 (d, *J* = 8.5 Hz, 1H, H-6’), 8.31 (d, *J* = 8.5 Hz, 1H, H-4’), 8.08 (s, 1H, N=CH), 7.81-7.78 (m, 2H, H-2 and H-6), 7.67 (t, *J* = 8.5 Hz, 1H, H-5’), 7.54-7.51 (m, 3H, H-3, H-4 and H-5); ^13^C-NMR (75 MHz, CDCl_3_): δ 148.5 (C-1), 148.1 (C-3’), 138.8 (C-6’), 132.0 (N=CH), 131.9 (C-1’), 130.4 (C-4), 129.6 (C-5’), 129.2 (C-3 and C-5), 124.7 (C-4’), 123.2 (C-2’), 121.5 (C-2 and C-6); EIMS (70 eV) m/z (% relative abundance): 242 (5) [M]^+·^, 141 (4) [M-1]+·, 226 (100) [M-O]^+^, 225 (50) [M-OH]^+·^.

**(*Z*)-*N*-(3,4,5-trimethoxybenzylidene) aniline oxide (3i).** 78% yield; yellow solid, Mp 187 °C; ^1^H-NMR (300 MHz, CDCl_3_): δ 7.88 (s, 1H, N=CH), 7.79 (s, 2H, H-2’ and H-6’), 7.79-7.76 (m, 2H, H-2 and H-6), 7.50-7.47 (m, 3H, H-3, H-4 and H-5), 3.94 (s, 9H, OMe); ^13^C-NMR (75MHz, CDCl_3_): δ 153.1 (C-5’ and C-3’), 149.1 (C-1), 140.4 (C-4’), 134.7 (N=CH), 130.1 (C-4), 129.4 (C-3 and C-5), 126.3 (C-1’), 121.8 (C-2 and C-6), 106.6 (C-2’ and C-6’), 61.2 (*p*-OMe), 56.3 (*m*-OMe); EIMS (70 eV) m/z (% relative abundance): 227(50) [M]^+·^, 226 (80) [M-1]^+^, 210 (100) [M-OH]^+^.

**(*Z*)-*N*-(2-methoxybenzylidene) aniline oxide (3j).** 80% yield; yellow solid, Mp 115 °C; ^1^H-NMR (200 MHz, CDCl_3_): δ 9.47 (dd, *J* = 8.4, 1.6, Hz, 1H, H-6’), 8.40 (s, 1H, N=CH), 7.81-7.76 (m, 2H, H-2 and H-6), 7.50-7.40 (m, 3H, H-3, H-4 and H-5), 7.13 (td, *J* = 8.4, 1.6 Hz, 1H, H-4’), 6.95 (m, 2H, H-3’ and H-5’), 3.88 (OMe); ^13^C-NMR (50 MHz, CDCl_3_): δ 157.4 (C-2’), 132.2 (N=CH), 129.6 (C-3 and C-5), 129.0 (C-4), 128.7 (C-6’), 128.4 (C-1’), 121.7 (C-2 and C-6), 120.7 (C-3’), 109.8 (C-5’), 55.5 (OCH_3_); EIMS (70 eV) m/z (% relative abundance): 227(50) [M]^+·^, 226 (80) [M-1]^+^, 210 (100) [M-OH]^+^.

**(*Z*)-*N*-(2,3-dihydroxybenzylidene) aniline oxide (3k).** 79% yield; yellow solid, Mp 210 °C; ^1^H-NMR (200 MHz, CDCl_3_): δ 8.10 (s, 1H, N=CH), 7.81-7.76 (m, 2H, H-2 and H-6), 7.55-7.51 (m, 3H, H-3, H-4 and H-5), 7.13 (dd, *J* = 7.6, 1.6 Hz, 1H, H-6’), 6.83 (t, *J* = 7.6, 1H, H-5’), 6.75 (dd, *J* = 7.6, 1.6, 1H, H-4’); ^13^C-NMR (50 MHz, CDCl_3_): δ 153.3 (C-2’), 148.0 (C-1), 141.3 (C-4), 130.6 (N=CH), 129.4 (C-3 and C-5), 123.2 (C-3’), 121.8 (C-2 and C-6), 119.8 (C-1’), 117.7 (C-5’), 115.9 (C-4’); EIMS (70 ev) m/z (% relative abundance): 198 (28) [M-OH]^+^, 169 (100) [M-CO_2_H]^+·^.

**Single-Crystal X-ray Crystallography.** A single crystal of compound **3i** was obtained by recrystallization from EtOH, as yellow crystals. These were mounted on glass fibers. Crystallographic measurements were performed using Mo KR radiation (graphite crystal monochromator, e = 71073 A°) at room temperature. Three standard reflections, which were monitored periodically, showed no change during data collection. Unit cell parameters were obtained from least-squares refinement of 26 reflections in the range 2 < 2e < 20. Intensities were corrected for Lorentz and polarization effects. No absorption correction was applied. Anisotropic temperature factors were introduced for all non-hydrogen atoms. Hydrogen atoms were placed in idealized positions and their atomic coordinates refined. Unit weights were used in the refinement. Structure was solved using the SHELX97 [25], and refined using SHELX97 on a personal computer.

## 4. Conclusions

An eco-friendly, novel and efficient synthesis of benzylideneaniline oxides is presented. The main advantages being: the achievement of good yields in short reaction times using microwave-assistance, and in the absence of solvent, and catalyst, together with appropriate atom economy and, moreover, the production of water as the only residuum product. According to our results, we suggest that this method displace other methods that use various organic solvents, catalysts and that are performed at high temperature.

## Figures and Tables

**Table 1:** Synthesis of **3a–k** with a green approach.

**Product**	**G**	**Melting point (°C)**	**Yield (%)**
**3a**	*m*-OH	95	76
**3b**	*o*-OH	147	85
**3c**	*p*-NO_2_	180	83
**3d**	*o*-NO_2_	85	77
**3e**	*p-*OCH_3_	108	86
**3f**	H	130	81
**3g**	*m*-NO_2_	110	68
**3h**	*p*-N(CH_3_)_2_	126	75
**3i**	3,4,5-triOCH_3_	187	78
**3j**	*o*-OCH_3_	115	80
**3k**	2,3-diOH	210	79
